# Tumor immune microenvironment and clinical outcomes in stage IV urothelial cancer: YODO study

**DOI:** 10.1007/s10147-023-02386-y

**Published:** 2023-07-27

**Authors:** Hiroyuki Nishiyama, Toyonori Tsuzuki, Chikara Ohyama, Hideyasu Matsuyama, Kenta Shinozaki, Yuko Hayashi, Nobuya Hayashi, Ryo Koto, Eisei Shin, Osamu Ogawa

**Affiliations:** 1 Department of Urology, University of Tsukuba, 2-1-1 Amakubo, Tsukuba, Ibaraki 305-8576 Japan; 2 Department of Surgical Pathology, Aichi Medical University, 1-1 Yazakokarimata, Nagakute, Aichi 480-1195 Japan; 3 Department of Urology, Hirosaki University, 5 Zaifu-Cho, Hirosaki, Aomori 036-8562 Japan; 4 Department of Urology, Graduate School of Medicine, Yamaguchi University, 1-1-1 Minamikogushi, Ube, Yamaguchi 755-8505 Japan; 5 AstraZeneca K.K, 3-1 Ofukacho, Kita-Ku, Osaka, 530-0011 Japan; 6 Department of Urology, Japanese Red Cross Otsu Hospital, 1-1-35 Nagara, Otsu, Shiga 520-8511 Japan

**Keywords:** B7-H1 antigen, Immune checkpoint inhibitors, Mortality, Tumor microenvironment, Urinary bladder neoplasms

## Abstract

**Background:**

Bladder cancer is the 10th most common cancer globally, with a growing incidence in Japan. Evaluation of molecular, genetic, and cellular biomarkers that predict treatment response and prognosis in patients with metastatic urothelial carcinoma (mUC) may help optimize sequential treatment strategies with chemotherapy and immune checkpoint inhibitors (ICIs).

**Methods:**

This multicenter, retrospective cohort study, evaluated programmed death-ligand 1 (PD-L1) expression, tumor mutational burden (TMB), and cancer-immune phenotype as predictive prognostic biomarkers following first-/second-line treatment in Japanese adult patients with mUC. The primary endpoint was prevalence of PD-L1 expression. Secondary endpoints were TMB, overall survival (OS), and progression-free survival (PFS) from initiation of first-line treatment, and exploratory endpoints were cancer-immune phenotype, OS, PFS, and treatment response according to potential biomarker status.

**Results:**

Of the 143 patients included (mean age 71.7 years), PD-L1 expression was high in 29.4% of patients. Non-synonymous TMB was high in 33.6% and low in 66.4%. Cancer-immune phenotype was immune-desert in 62.9%, immune-excluded in 30.8%, and inflamed in 6.3%. Median OS and PFS following first-line treatment were 18.2 and 7.4 months, respectively. Overall response to second-line treatment was slightly better with high versus low/negative PD-L1 expression. PD-L1 expression and TMB were non-significant predictors of OS or PFS, whereas immune-excluded phenotype was associated with better OS in comparison with immune-desert phenotype.

**Conclusion:**

PD-L1 expression and TMB were non-significant predictors of prognosis after first-line treatment in Japanese patients with mUC, but cancer-immune phenotype may be an important prognostic factor in chemotherapy-ICI sequential treatment strategies.

*Clinical trial registration number* UMIN000037727.

**Supplementary Information:**

The online version contains supplementary material available at 10.1007/s10147-023-02386-y.

## Introduction

Bladder cancer is the 10^th^ most common type of cancer globally, with an incidence of three per 100,000 persons worldwide [1, 2]. The incidence of bladder cancer in Japan is growing with a total of 23,383 new cases reported in 2019, of which 90% were urothelial carcinoma (UC) [3, 4]. The 5-year survival rate of patients with stage I to stage IV UC decreases from 97 to 22% [4].

Clinical practice guidelines in Japan [5] and Europe [6] recommend cisplatin-based combinations as first-line treatment for cisplatin-eligible patients with metastatic UC (mUC) and carboplatin for cisplatin-ineligible patients. Treatment options for the latter group have increased with the availability of immune checkpoint inhibitors (ICIs). However, pembrolizumab and avelumab are the only ICIs available for the treatment of mUC in Japan; pembrolizumab was approved as second-line treatment and avelumab as maintenance therapy [7–9].

ICIs prevent tumor immune tolerance by blocking the programmed cell death protein 1 (PD-1) and its ligand (PD-L1). Pembrolizumab as second-line therapy for advanced UC demonstrated a limited overall response rate (ORR; i.e., complete response [CR] or partial response [PR]) of 21.1% [10]. While durable responses can sometimes be achieved, some patients experience disease progression during the therapy [11]. Predictive biomarkers of sensitivity and resistance to ICIs are needed to identify optimal treatment for these patients. A systematic review found that the most common biomarker, PD-L1, only predicted treatment response in 28.9% of studies investigating United States Food and Drug Administration-approved ICIs [12]. Tumor mutational burden (TMB) has also emerged as a potential predictive biomarker in patients with UC [13] and mUC [14]. Combination use of TMB and PD-L1 can be used to define the immunologic state of the tumor microenvironment, potentially predicting treatment response to ICIs. A study of 9,887 clinical samples showed that low PD-L1 expression and a median TMB of < 10 mutations/Mb were characteristic of non-inflamed tumor types which were unlikely to respond to ICIs [15]. Cancer-immune phenotype can also be a predictive biomarker for treatment response and prognosis. Patients with immune-excluded and immune-desert phenotypes are known to show poor response to ICIs [16, 17]. Collectively, evaluation of these factors may help optimize therapeutic strategies for patients with mUC.

The aim of this study was to evaluate patterns of the tumor immune microenvironment including PD-L1 expression, TMB, and cancer-immune phenotype, and their prognostic implication among Japanese patients with stage IV UC who received first-line chemotherapy and second-line ICIs or chemotherapy.

## Patients and methods

### Study design and patients

This retrospective cohort study was conducted in 21 centers in Japan between October 2019 and March 2020, and enrolled stage IV UC patients (aged ≥ 20 years) who had received ≥ 1 cycle of chemotherapy (not ICI) as first-line treatment. Eligible patients were diagnosed with stage IV UC according to the American Joint Committee on Cancer [18] between 1 January 2017 and 31 December 2018, and had formalin-fixed paraffin-embedded (FFPE) primary tumor samples collected in the 3 years before the start of first-line treatment. All patients included in the study provided written informed consent prior to study participation. In patients who had died or were lost to follow-up prior to registration, opt-out was applicable according to the ethical committee/institutional review board’s approval. Patient data were retrospectively collected from medical records at each site and archival tissue samples were sent to a central laboratory (Riken Genesis Co., Ltd.) for analysis.

The study protocol was approved by an independent ethics committee (Non-Profit Organization MINS Institutional Review Board, Approval ID: 190235). The study was reviewed and approved by the ethics committee at each site under the guidelines of the Declaration of Helsinki and the Ethical Guidelines for Medical and Health Research Involving Human Subjects (promulgated on 22 December 2014 and partially revised on 28 February 2017).

### Study endpoints

The primary endpoint was the prevalence of PD-L1 expression in tumor and immune cells, including the proportion of patients with high and low/negative PD-L1 expression. High PD-L1 expression was defined as expression in ≥ 25% of tumor cells; or > 1% immune cell presence and expression in ≥ 25% of immune cells; or 1% immune cell presence and expression in 100% of immune cells. All other patients were classified as having low/negative PD-L1 expression [19].

The key secondary endpoint was the proportion of patients with high and low TMB (including any TMB and non-synonymous TMB). High TMB was defined as ≥ 10 mutations/Mb and low TMB was < 10 mutations/Mb. Other secondary endpoints were overall survival (OS; time from initiation of first-line treatment to death or lost to follow-up) and progression-free survival (PFS; time from initiation of first-line treatment to disease progression or death).

Exploratory endpoints were the prevalence of different cancer-immune phenotypes, including immune-desert (defined and characterized by the absence of tumor infiltrating lymphocytes [TILs] within the tumor or its periphery), immune-excluded (defined by the accumulation of TILs around the tumor with infiltration limited to the tumor), and inflamed (defined by TILs accumulating in the entire tumor area including its periphery) [20]; OS, PFS, and response to first- and second-line treatment in the various patient subpopulations.

### Study measurements

Patient characteristics and pre-therapy laboratory data were collected from medical records.

Primary tumor FFPE samples were collected from each site for assessment of PD-L1 expression, TMB, and cancer-immune phenotype. Pathologic diagnosis of UC and cancer-immune phenotype was assessed by two pathologists (Dr. T. Tsuzuki and Dr. S. Mikami) from the central pathology committee. PD-L1 expression was assessed by immunohistochemistry with SP263 antibodies by SRL Diagnostics (Tokyo, Japan), and TMB was assessed by next-generation sequencing at Riken Genesis Co., Ltd. using commercially available methods (TruSight Oncology 500 DNA Kit and TruSight Oncology 500 Local App; Illumina, Tokyo, Japan). Based on these data, categories of PD-L1 expression, TMB, and the cancer-immune phenotypes were determined.

Response to treatment was assessed as CR, PR, stable disease, or progressive disease according to Response Evaluation Criteria in Solid Tumors, version 1.1 by the site investigators.

### Statistical analysis

The primary endpoint was the prevalence of high or low/negative PD-L1 expression and the key secondary endpoint was the prevalence of high or low TMB summarized as the number of patients and calculated percentages.

Median OS and PFS were estimated based on the Kaplan–Meier method and are presented with their respective 95% confidence intervals (CIs). Cox regression model was used to calculate hazard ratios (HRs) and 95% CIs for comparisons of OS and PFS between PD-L1 expression (high vs. low/negative), non-synonymous TMB (low vs. high), and cancer-immune phenotype (immune-excluded or inflamed vs. immune-desert) subgroups. Univariate analysis was conducted to evaluate which patient covariates were potentially associated with OS.

Statistical analysis was performed using SAS Enterprise Guide software version 9.4 or higher (SAS Institute Inc. SAS/STAT, Cary, NC).

## Results

### Study population

The full analysis set included 143 patients with stage IV UC (Supplementary Fig S1; Online Resource 1). Patient characteristics are summarized in Table 1. The mean (standard deviation) age was 71.7 (9.8) years; most patients were male (70.6%) and had a diagnosis of bladder cancer (76.2%). Sixty-three patients (44.1%) had previous surgery and 40 (28.0%) had previous radiotherapy.

**Table 1:** Patient characteristics of the study cohort

	All (N = 143)	Creatinine clearance, mL/min	
		< 60 (n = 86)	≥ 60 (n = 50)
*Patient baseline characteristics*			
Status of enrolment, n (%)			
Alive	60 (42.0)	28 (32.6)	28 (56.0)
Opt-out			
Death	79 (55.2)	55 (64.0)	21 (42.0)
Lost to follow-up	4 (2.8)	3 (3.5)	1 (2.0)
Sex, n (%)			
Male	101 (70.6)	60 (69.8)	35 (70.0)
Female	42 (29.4)	26 (30.2)	15 (30.0)
Age, mean (SD), years	71.7 (9.8)	76.1 (7.0)	64.0 (9.8)
BMI, kg/m^2^			
No. of patients	129	78	48
Mean (SD)	22.6 (3.3)	21.9 (2.8)	23.7 (3.8)
Missing	14	8	2
Smoking status, n (%)			
Current smoker	20 (14.0)	10 (11.6)	10 (20.0)
Ex-smoker	57 (39.9)	33 (38.4)	21 (42.0)
Never smoked	64 (44.8)	43 (50.0)	18 (36.0)
Missing/unknown	2 (1.4)	0	1 (2.0)
UC diagnosis^a^, n (%)			
Bladder cancer	109 (76.2)	56 (65.1)	47 (94.0)
Pelvic/ureteral cancer	46 (32.2)	40 (46.5)	5 (10.0)
Any metastasis at stage IV UC diagnosis, n (%)			
Yes	133 (93.0)	80 (93.0)	46 (92.0)
No	8 (5.6)	4 (4.7)	4 (8.0)
Missing/unknown	2 (1.4)	2 (2.3)	0
Metastasis site at stage IV UC diagnosis^a^, n (%)			
Lymph node	113 (79.0)	72 (83.7)	35 (70.0)
Lung	31 (21.7)	19 (22.1)	11 (22.0)
Other^b^	26 (18.2)	15 (17.5)	11 (22.0)
ECOG performance status, n (%)			
0	40 (28.0)	24 (27.9)	15 (30.0)
1	25 (17.5)	19 (22.1)	6 (12.0)
≥ 2	4 (2.8)	3 (3.5)	1 (2.0)
Missing/unknown	74 (51.7)	40 (46.5)	28 (56.0)
Creatinine clearance, n (%)			
< 60 mL/min	86 (60.1)	86 (100.0)	–
≥ 60 mL/min	50 (35.0)	–	50 (100.0)
Missing/unknown	7 (4.9)	–	–
Neutrophil-to-lymphocyte ratio, n (%)			
< 500	104 (72.7)	64 (74.4)	39 (78.0)
≥ 500	24 (16.8)	14 (16.3)	9 (18.0)
Missing/unknown	15 (10.5)	8 (9.3)	2 (4.0)
Previous surgery, n (%)			
Yes	63 (44.1)		
No	80 (55.9)		
Previous radiotherapy, n (%)			
Yes	40 (28.0)		
No	103 (72.0)		
Surgery for tumor removal, n (%)			
Radical cystectomy	29 (20.3)	16 (18.6)	12 (24.0)
Radical nephroureterectomy	27 (18.9)	25 (29.1)	1 (2.0)
Other^c^	87 (60.8)	45 (52.3)	37 (74.0)
*Characteristics of FFPE samples*			
Anatomical site, n (%)			
Bladder	106 (74.1)	53 (61.6)	47 (94.0)
Renal pelvis	29 (20.3)	26 (30.2)	2 (4.0)
Ureter	8 (5.6)	7 (8.1)	1 (2.0)
Urethra	0	0	0
Surgical procedure for removal, n (%)			
Radical cystectomy	29 (20.3)	16 (18.6)	12 (24.0)
Radical nephroureterectomy	27 (18.9)	25 (29.1)	1 (2.0)
Transurethral resection of cancer lesions	76 (53.1)	38 (44.2)	33 (66.0)
Partial cystectomy	1 (0.7)	0	1 (2.0)
Unknown	10 (7.0)	7 (8.1)	3 (6.0)
Histology^d^, n (%)			
UC	143 (100.0)	86 (100.0)	50 (100.0)
Variant, n (%)			
Pure	110 (76.9)	67 (77.9)	36 (72.0)
Variant	33 (23.1)	19 (22.1)	14 (28.0)
Muscle invasive cancer, n (%)			
Yes	109 (76.2)	70 (81.4)	34 (68.0)
No	32 (22.4)	16 (18.6)	14 (28.0)
Missing/unknown	2 (1.4)	0	2 (4.0)
*Treatment*			
First-line chemotherapy regimen, n (%)			
GC^e^	81 (56.7)	35 (40.7)	40 (80.0)
GCa	48 (33.6)	43 (50.0)	4 (8.0)
MVAC or DD-MVAC	0	0	0
Other	14 (9.8)	8 (9.3)	6 (12.0)
≥ 2 lines of therapy, n (%)			
Any chemotherapy	100 (70.0)	57 (66.2)	37 (74.0)
Pembrolizumab^f^	89 (62.2)	52 (60.5)	32 (64.0)
Perioperative neoadjuvant chemotherapy, n (%)			
Yes	22 (15.4)	11 (12.8)	11 (22.0)
No	121 (84.6)	75 (87.2)	39 (78.0)
Perioperative adjuvant chemotherapy, n (%)			
Yes	3 (2.1)	1 (1.2)	1 (2.0)
No	140 (97.9)	85 (98.8)	49 (98.0)

*BMI* body mass index, *DD-MVAC* dose-dense methotrexate + vinblastine + doxorubicin + cisplatin, *ECOG* Eastern Cooperative Oncology Group*, FFPE* formalin-fixed paraffin-embedded*, GC* gemcitabine + cisplatin*, GCa* gemcitabine + carboplatin*, MVAC* methotrexate + vinblastine + doxorubicin + cisplatin*, SD* standard deviation*, UC* urothelial carcinoma

^a^Includes multiple overlapping responses; ^b^Includes metastases in bone, peritoneum, liver, and pleura; ^c^Includes transurethral resection of cancer lesion and partial cystectomy; ^d^According to central pathologic committee; ^e^Includes 4-week and 3-week GC; ^f^Includes patients who received pembrolizumab in any line (≥ second line)

Creatinine clearance was < 60 mL/min in 86 patients (60.1%) and ≥ 60 mL/min in 50 patients (35.0%). Of the 143 patients included, 81 (56.7%) received first-line chemotherapy with gemcitabine plus cisplatin (GC). FFPE bladder samples were collected from 106 patients (74.1%), renal pelvis samples from 29 (20.3), and ureter samples from eight (5.6%). Samples were collected by radical cystectomy in 29 patients (20.3%), radical nephroureterectomy in 27 (18.9%), transurethral resection of cancer lesions in 76 (53.1%), and partial cystectomy in one (0.7%); the methods of collection for the remaining 10 (7.0%) samples were unknown. Histologically, all samples were consistent with UC, including muscle invasive in 109 samples (76.2%) and non-muscle invasive in 32 (22.4%); histology information of tumor depth was missing for two samples (1.4%). First-line treatment with gemcitabine plus carboplatin was administered in 48/143 patients (33.6%), of which 43 patients (91.5%) had creatinine clearance < 60 mL/min. Second-line or later treatment was administered in 100 patients (70%). A total of 89 patients (62.2%) received immunotherapy with pembrolizumab.

### PD-L1 expression

Central pathologic review of FFPE samples showed that tumor PD-L1 expression was high in 42 patients (29.4%) and low/negative in 101 patients (70.6%; Table 2). Twenty-six patients (18.2%) had high PD-L1 expression according to the first immune cell definition (> 1% immune cell presence and PD-L1 expression in ≥ 25% of immune cells); none had high PD-L1 expression according to the second definition (1% immune cell presence and PD-L1 expression in 100% of immune cells).

**Table 2:** Programmed death-ligand 1 expression and its distribution by prevalence of non-synonymous tumor mutational burden and cancer-immune phenotype

	All (N = 143)	Non-synonymous TMB		Cancer-immune phenotype		
		High (n = 48)	Low (n = 95)	Immune-desert (n = 90)	Immune-excluded (n = 44)	Inflamed (n = 9)
PD-L1 expression, n (%)*						
High	42 (29.4)	17 (35.4)	25 (26.3)	11 (12.2)	23 (52.3)	8 (88.9)
Low/negative	101 (70.6)	31 (64.6)	70 (73.7)	79 (87.8)	21 (47.7)	1 (11.1)

*PD-L1* programmed death-ligand 1, *TMB* tumor mutational burden

### TMB prevalence and cancer-immune phenotype

The prevalence of any TMB was high in approximately half of the study population (50.3%; n = 72), whereas the prevalence of non-synonymous TMB was high in approximately one-third of patients (33.6%; n = 48) and low in 66.4% of patients (n = 95; Table 2). PD-L1 expression was low/negative in most patients with high (64.6%; n = 31) or low (73.7%; n = 70) non-synonymous TMB.

Cancer-immune phenotype was immune-desert in 90 patients (62.9%), immune-excluded in 44 (30.8%), and inflamed in nine patients (6.3%). PD-L1 expression was low/negative in most patients with an immune-desert phenotype (87.8%; n = 79), but high in over half the patients with an immune-excluded phenotype (52.3%; n = 23) and most patients with an inflamed phenotype (88.9%; n = 8; Table 2). Immune-excluded phenotype was the most prevalent among patients with high PD-L1 expression (54.8%; n = 23), while immune-desert was the most prevalent among those with low/negative PD-L1 expression (78.2%; n = 79). Immune-desert was also the most prevalent phenotype among patients with high or low non-synonymous TMB (Supplementary Table S1; Online Resource 1).

Pre-therapy laboratory data and prognostic covariates are summarized in Supplementary Tables S2–S4 (Online Resource 1).

### Survival analysis

**Fig. 1:**
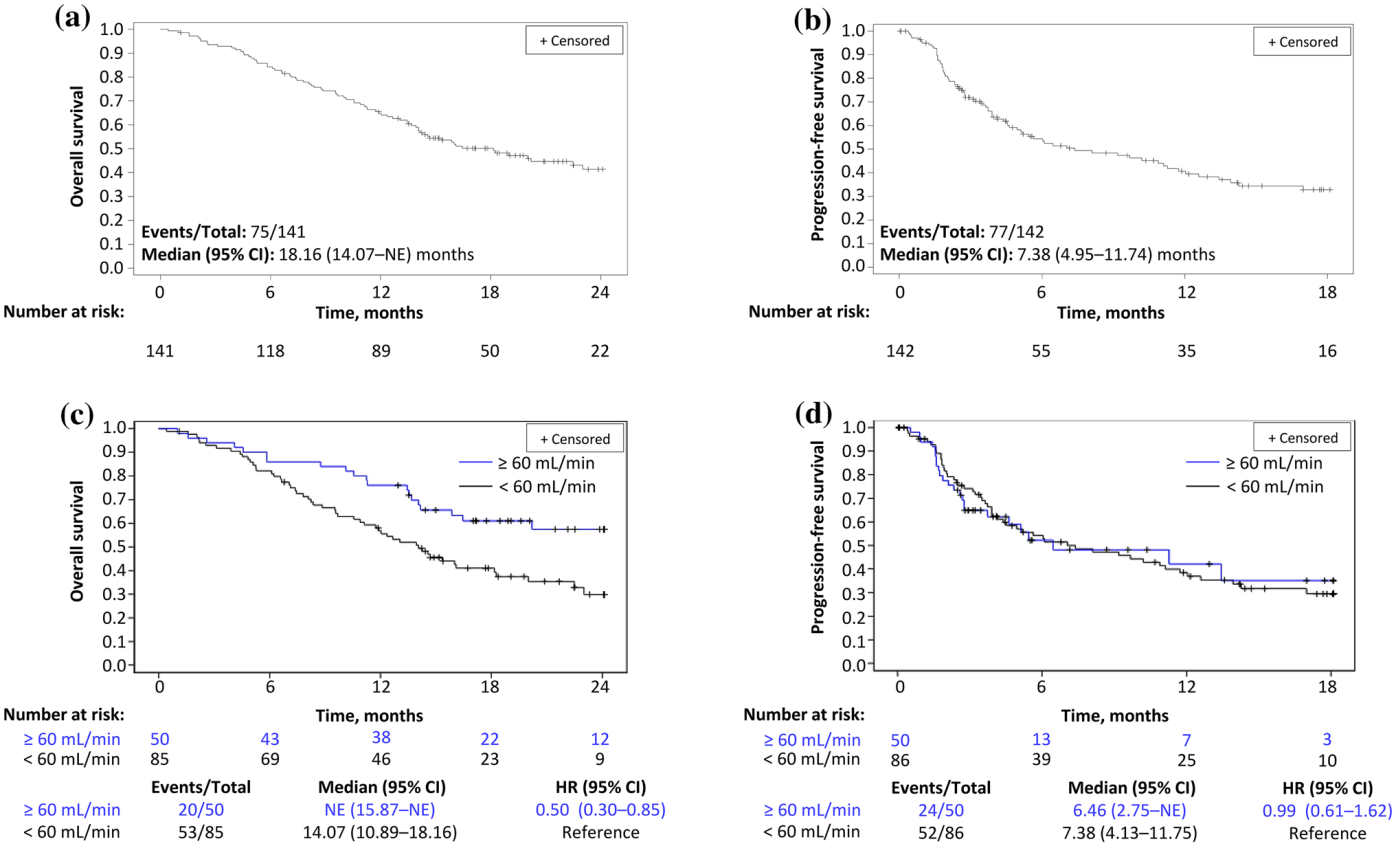
Kaplan–Meier curves showing overall survival and progression-free survival in the overall population (**a**, **b**, respectively) and overall survival and progression-free survival by pre-treatment renal function (**c**, **d**, respectively). *CI* confidence interval, *HR* hazard ratio, *NE* not evaluable

Overall, median OS was 18.2 (95% CI 14.1–not evaluable [NE]) months (Fig. 1a) and median PFS was 7.4 (95% CI 5.0–11.7) months (Fig. 1b). The OS rate was 64.9% at 12 months, 50.2% at 18 months, and 41.4% at 24 months, and the PFS rate at 6, 12 and 18 months was 54.4%, 40.7%, and 32.9%, respectively.

When assessed by renal function, median OS was 14.1 (95% CI 10.9–18.2) months in patients with creatinine clearance < 60 mL/min and NE in those with creatinine clearance ≥ 60 mL/min (Fig. 1c). Median PFS was 7.4 (95% CI 4.1–11.7) months and 6.5 (95% CI 2.8–NE) months in the respective subgroups (Fig. 1d).

**Fig. 2:**
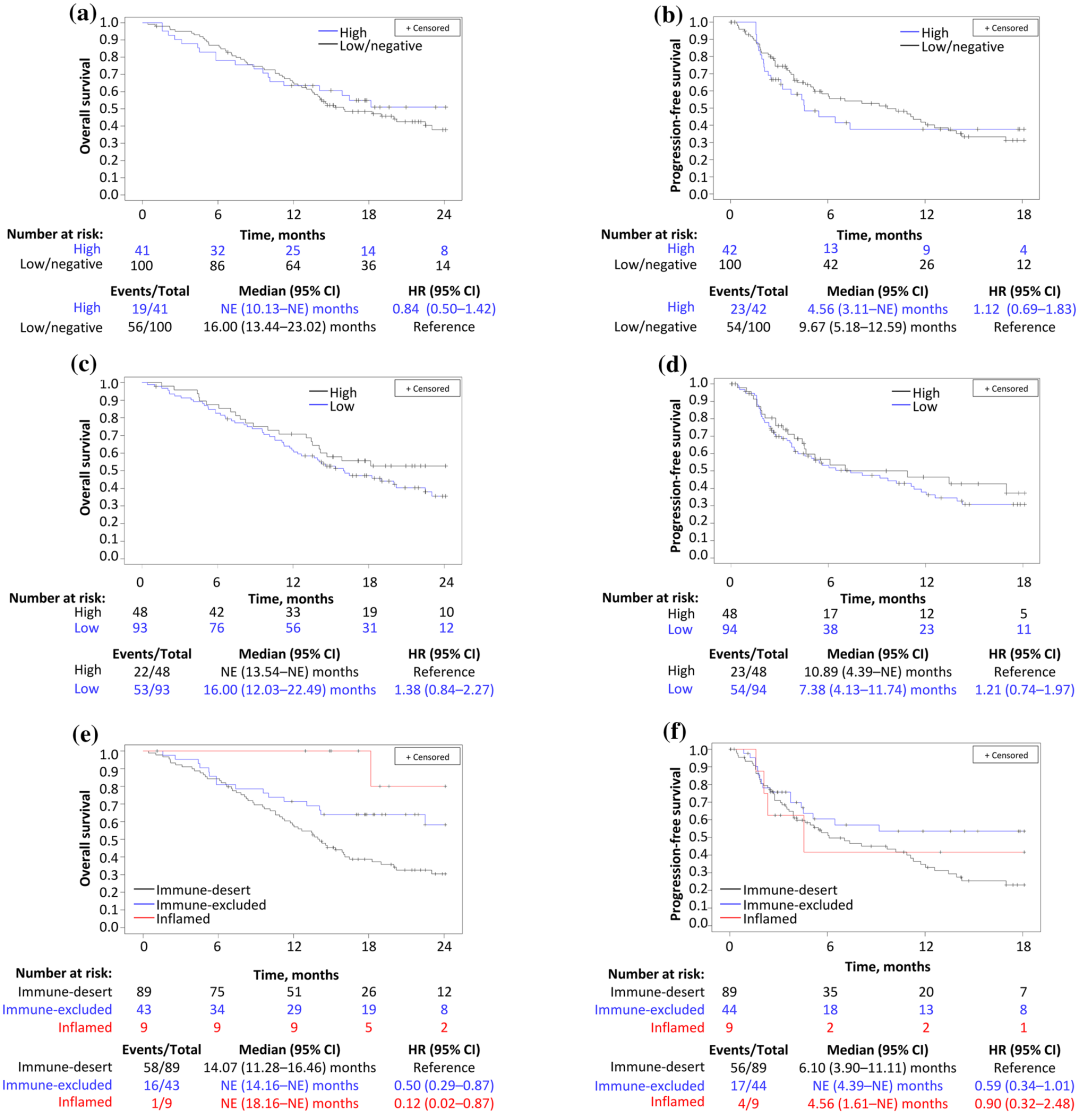
Kaplan–Meier curves showing overall survival and progression-free survival by programmed death-ligand 1 expression (**a**, **b**, respectively), overall survival and progression-free survival by non-synonymous tumor mutational burden (**c**, **d**, respectively), and overall survival and progression-free survival by cancer-immune phenotype (**e**, **f**, respectively). *CI* confidence interval, *HR* hazard ratio, *NE* not evaluable

In the survival analysis by PD-L1 expression, median OS was 16.0 (95% CI 13.4–23.0) months with low/negative PD-L1 expression and NE with high PD-L1 expression (Fig. 2a). Median PFS was 9.7 (95% CI 5.2–12.6) months with low/negative PD-L1 expression and 4.6 (95% CI 3.1–NE) months with high PD-L1 expression (Fig. 2b). There was no statistically significant difference for high versus low/negative PD-L1 expression for OS (HR 0.84; 95% CI 0.50–1.42) or PFS (HR 1.12; 95% CI 0.69–1.83).

By non-synonymous TMB, median OS was 16 (95% CI 12.0–22.5) months with low non-synonymous TMB and NE with high non-synonymous TMB (Fig. 2c). Median PFS was 7.4 (95% CI 4.1–11.7) months with low non-synonymous TMB and 10.9 (95% CI 4.4–NE) months with high non-synonymous TMB (Fig. 2d). There was also no significant difference between the low versus high non-synonymous TMB groups for OS (HR 1.38; 95% CI 0.84–2.27) or PFS (HR 1.21; 95% CI 0.74–1.97).

By cancer-immune phenotype, median OS was 14.1 (95% CI 11.3–16.5) months in patients with the immune-desert phenotype, and NE in those with the immune-excluded and inflamed phenotypes, respectively (Fig. 2e). Median PFS was 6.1 (95% CI, 3.9–11.1) months, NE, and 4.6 (95% CI, 1.6–NE) months in the immune-desert, immune-excluded, and inflamed phenotypes, respectively (Fig. 2f). In this analysis, the risk of death was significantly lower in the immune-excluded versus immune-desert phenotype (HR 0.50; 95% CI 0.29–0.87).

### Treatment response

After first-line chemotherapy, eight patients (5.6%) achieved CR, and 21 (14.7%) achieved PR (Table 3). First-line response was slightly better in patients with low/negative PD-L1 expression with an ORR of 21.7% compared with 16.7% in patients with high PD-L1 expression. In contrast, ORR was better in patients with high non-synonymous TMB (25.0%) than in patients with low non-synonymous TMB (17.9%). Among patients treated with first-line chemotherapy, the ORR was better in patients with the inflamed phenotype (33.3%), than the immune-desert (16.7%), and immune-excluded (25%) phenotypes, whereas patients with the immune-excluded phenotype had the highest disease control rate (DCR; 54.4%; Table 3).

**Table 3:** Overall survival and response to first- and second-line treatment by programmed death-ligand 1 expression, tumor mutational burden, and cancer-immune phenotype

	All (N = 143)	PD-L1 expression		Non-synonymous TMB		Cancer-immune phenotype		
		High (n = 42)	Low/Negative (n = 101)	High (n = 48)	Low (n = 95)	Immune-desert (n = 90)	Immune-excluded (n = 44)	Inflamed (n = 9)
*Event*								
Death from deterioration or complications of primary disease	66 (46.2)	49 (48.5)	17 (40.5)	17 (35.4)	49 (51.6)	53 (58.9)	12 (27.3)	1 (11.1)
Death from causes unrelated to progression of primary disease	4 (2.8)	3 (3.0)	1 (2.4)	1 (2.1)	3 (3.2)	3 (3.3)	1 (2.3)	0
Disease progression	55 (38.5)	38 (37.6)	17 (40.5)	16 (33.3)	39 (41.1)	37 (41.1)	14 (31.8)	4 (44.4)
Death from unknown causes	7 (4.9)	5 (5.0)	2 (4.8)	4 (8.3)	3 (3.2)	3 (3.3)	4 (9.1)	0
Median OS (95% CI)	18.2 (14.1–NE)	16.0 (13.4–23.0)	NE (10.1–NE)	NE (13.5–NE)	16.0 (12.0–22.5)	14.1 (11.3–16.5)	NE (14.2–NE)	NE (18.2–NE)
Median PFS (95% CI)	7.4 (5.0–11.7)	9.7 (5.2–12.6)	4.6 (3.1–NE)	10.9 (4.4–NE)	7.4 (4.1–11.7)	6.1 (3.9–11.1)	NE (4.4–NE)	4.6 (1.6–NE)
*First-line response*^*a*^								
CR	8 (5.6)	2 (4.8)	6 (5.9)	1 (2.1)	7 (7.4)	5 (5.6)	2 (4.5)	1 (11.1)
PR	21 (14.7)	5 (11.9)	16 (15.8)	11 (22.9)	10 (10.5)	10 (11.1)	9 (20.5)	2 (22.2)
SD	31 (21.7)	7 (16.7)	24 (23.8)	10 (20.8)	21 (22.1)	18 (20.0)	13 (29.5)	0
PD	59 (41.3)	18 (42.9)	41 (40.6)	18 (37.5)	41 (43.2)	39 (43.3)	16 (36.4)	4 (44.4)
NE	24 (16.8)	10 (23.8)	14 (13.9)	8 (16.7)	16 (16.8)	18 (20.0)	4 (9.1)	2 (22.2)
*Second-line response*^*a*^	(n = 100)	(n = 30)	(n = 70)	(n = 34)	(n = 66)	(n = 63)	(n = 29)	(n = 8)
CR	8 (8.0)	4 (13.3)	4 (5.7)	6 (17.6)	2 (3.0)	4 (6.3)	1 (3.4)	3 (37.5)
PR	11 (11.0)	4 (13.3)	7 (10.0)	5 (14.7)	6 (9.1)	5 (7.9)	6 (20.7)	0
SD	20 (20.0)	6 (20.0)	14 (20.0)	5 (14.7)	15 (22.7)	10 (15.9)	7 (24.1)	3 (37.5)
PD	49 (49.0)	13 (43.3)	36 (51.4)	16 (47.1)	33 (50.0)	37 (58.7)	11 (37.9)	1 (12.5)
NE	9 (9.0)	3 (10.0)	6 (8.6)	2 (5.9)	7 (10.6)	4 (6.3)	4 (13.8)	1 (12.5)
Missing	3 (3.0)	0	3 (4.3)	0	3 (4.5)	3 (4.8)	0	0

*CI* confidence interval, *CR* complete response, *NE* not evaluable, *OS* overall survival, *PFS* progression-free survival*, PD* progressive disease, *PD-L1* programmed death-ligand 1, *PR* partial response, *RECIST* Response Evaluation Criteria in Solid Tumors, *SD* stable disease, *TMB* tumor mutational burden

^a^Assessed by primary and secondary investigators

Among the 100 patients with second-line treatment, eight (8.0%) achieved CR and 11 (11.0%) achieved PR (Table 3). Following second-line treatment, the ORR was 26.6% with high PD-L1 expression, and 15.7% with low/negative PD-L1 expression. ORR was 32.3% and 12.1% in patients with high versus low non-synonymous TMB, respectively. ORR and DCR were highest (37.5% and 75%, respectively) in patients with the inflamed phenotype (Table 3).

### Baseline patient characteristics/covariates associated with survival

Univariate analyses were conducted for OS (Table 4). The covariates with increased risk of death were Eastern Cooperative Oncology Group performance status of 1, creatinine clearance < 60 mL/min, serum albumin < 3.5 g/dL, C-reactive protein > 1 mg/L, white blood cells ≥ 8000/μL, a neutrophil–lymphocyte ratio ≥ 500, a platelet lymphocyte ratio ≥ 30,000, a prognostic index score of 1 or 2, and a modified Glasgow prognostic score of 1 or 2. The only covariate with a reduced risk of death was the immune-excluded phenotype.

**Table 4:** Univariate analysis of patient covariates potentially associated with overall survival following first-line treatment

Factor	N	Event, n (%)	OS^a^	
			Median (95% CI)	HR (95% CI)
*Age, years*				
< 65	28	11 (39.3)	NE (13.7–NE)	Reference
≥ 65–≤ 74	56	32 (57.2)	19.0 (11.3–NE)	1.8 (0.9–3.5)
> 74	59	34 (57.7)	14.6 (11.1–NE)	1.9 (1.0–3.8)
*Sex*				
Male	101	50 (49.5)	20.2 (14.1–NE)	Reference
Female	42	27 (64.3)	14.6 (8.8–22.5)	1.5 (0.9–2.4)
*ECOG performance status*				
0	40	13 (32.5)	NE (20.2–NE)	Reference
1	25	18 (72.0)	**11.7 (5.0–22.5)**	**3.4 (1.7–7.1)**
2	1	1 (100)	16.0 (NE–NE)	3.2 (0.4–24.5)
3	3	3 (100)	11.1 (2.1–13.7)	6.2 (1.7–22.4)
*Creatinine clearance, mL/min*				
≥ 60	50	20 (40.0)	NE (15.9–NE)	Reference
< 60	86	54 (62.9)	**14.1 (10.9–18.2)**	**2.0 (1.2–3.3)**
*Hemoglobin, g/dL*				
≥ 10	112	59 (52.8)	18.2 (14.1–NE)	Reference
< 10	25	16 (64.0)	11.2 (7.0–NE)	1.5 (0.9–2.6)
*Serum albumin, g/dL*				
≥ 3.5	87	41 (47.0)	22.5 (15.3–NE)	Reference
< 3.5	43	31 (52.1)	**10.6 (7.4–13.4)**	**2.2 (1.4–3.5)**
*C-reactive protein, mg/L*				
≤ 1	84	37 (44.1)	23.0 (16.5–NE)	Reference
> 1	44	34 (77.2)	**9.5 (5.1–11.7)**	**3.0 (1.9–4.8)**
*WBC count, /μL*				
< 8,000	100	46 (46.0)	22.5 (16.0–NE)	Reference
≥ 8,000	37	29 (78.4)	**10.3 (5.1–14.3)**	**2.5 (1.5–3.9)**
*NLR*				
< 500	104	49 (47.1)	22.5 (15.9–NE)	Reference
≥ 500	24	20 (83.3)	**5.5 (3.9–10.6)**	**3.4 (2.0–5.8)**
*PLR*				
< 15,000	50	21 (42.0)	NE (16.5–NE)	Reference
≥ 15,000–< 30,000	60	34 (56.7)	14.7 (11.3–23.0)	1.7 (1.0–3.0)
≥ 30,000	18	14 (77.9)	**6.5 (4.8–13.0)**	**3.5 (1.8–7.0)**
*Prognostic index*				
0	84	37 (44.1)	23.0 (16.5–NE)	Reference
1	32	24 (75.1)	**9.5 (5.1–12.4)**	**2.8 (1.6–4.7)**
2	13	10 (76.9)	**10.1 (1.6–13.0)**	**3.7 (1.9–7.2)**
*Prognostic nutrition index*				
0	130	74 (57.0)	15.3 (13.0–22.5)	Reference
1	5	1 (20.0)	NE (14.1–NE)	0.2 (0.0–1.7)
*mGPS*				
0	84	37 (44.1)	23.0 (16.5–NE)	Reference
1	14	10 (71.4)	**11.3 (2.6–NE)**	**2.2 (1.1–4.6)**
2	28	22 (78.6)	**8.3 (4.6–11.7)**	**3.3 (1.9–5.6)**
*Variant histology*				
Pure	109	58 (53.2)	18.2 (14.1–NE)	Reference
Variant	32	17 (53.1)	14.2 (9.6–NE)	1.1 (0.6–1.8)
*PD-L1 expression*				
Low/negative	100	56 (56.0)	16.0 (13.4–23.0)	Reference
High	41	19 (46.3)	NE (10.1–NE)	0.8 (0.5–1.4)
*Non-synonymous TMB*				
High	48	22 (45.8)	NE (13.5–NE)	Reference
Low	95	55 (57.9)	16.0 (12.0–22.5)	0.7 (0.4–1.2)
*Cancer-immune phenotype*				
Immune-desert	89	58 (65.2)	14.1 (11.3–16.5)	Reference
Immune-excluded	43	16 (37.2)	**NE (14.2–NE)**	**0.5 (0.3–0.9)**
Inflamed	9	1 (11.1)	NE (18.2–NE)	0.2 (0.0–0.9)

*CI* confidence interval*, ECOG* Eastern Cooperative Oncology Group, *HR* hazard ratio, *mGPS* modified Glasgow Prognostic Score, *NE* not evaluable, *NLR* neutrophil-to-lymphocyte ratio, *OS* overall survival, *PD-L1* programmed death-ligand 1, *PLR* platelet-to-lymphocyte ratio, *TMB* tumor mutational burden, *WBC* white blood cell

^a^Parameters with ≥ 10 events with 95% CI not including 1 are presented in bold

## Discussion

This study described the tumor microenvironment of stage IV UC in Japanese patients in relation to survival outcomes. High PD-L1 expression was found in 29.4% of patients and high non-synonymous TMB was identified in 33.6% of patients. The most prevalent cancer-immune phenotype was immune-desert (62.9%). PD-L1 expression and non-synonymous TMB were non-significant predictors of OS and PFS. OS was improved in patients with the immune-excluded phenotype (30.8%). Response to first-line chemotherapy was slightly better in patients with low/negative versus high PD-L1 expression, while response to second-line chemotherapy was better in patients with high PD-L1 expression; most patients receiving second-line treatment were treated with pembrolizumab. These findings are similar to previous studies in mUC showing an improved response to atezolizumab in patients with high PD-L1 expression (≥ 5% in tumor-infiltrating immune cells) [21, 22].

As pembrolizumab is approved as second-line or later treatment for mUC in Japan [7, 8], pembrolizumab was not available for 30% of our patients due to treatment line restriction. Some studies have addressed the efficacy of ICIs as first-line treatment. The phase 3 KEYNOTE-045 study was the only randomized clinical trial (RCT) to show improved outcomes with pembrolizumab monotherapy versus chemotherapy in platinum-ineligible patients with advanced UC, with a median OS of 10.3 (95% CI 8.0–11.8) months with pembrolizumab and 7.4 (95% CI 6.1–8.3) months with chemotherapy (*p* = 0.002) [11]. However, this study found no significant difference in PFS. The phase 3 IMvigor 130 RCT showed significant prolongation of PFS in patients with mUC who received atezolizumab plus platinum-based chemotherapy (8.2 [95% CI 6.5–8.3] months) versus chemotherapy alone (6.3 [95% CI 6.2–7.0] months; *p* = 0.007) [23]. In the phase 3 IMvigor 211 RCT, median OS was not significantly different between atezolizumab (11.1 [95% CI 8.6–15.5] months) and chemotherapy (10.6 [95% CI 8.4–12.2] months; *p* = 0.41) in patients with PD-L1 positive tumors [24]. Furthermore, the phase 3 KEYNOTE-361 [25] and DANUBE [26] RCTs showed lower ORRs for ICI monotherapy than for ICI/chemotherapy or chemotherapy alone. With these inconsistent results, there is a need for more robust biomarkers to predict treatment outcomes with ICIs.

Based on our results, PD-L1 expression and TMB were not prognostic, but cancer-immune phenotype was useful in predicting better OS in Japanese patients with stage IV UC. Combined with previous findings, the cancer-immune phenotype may be a useful biomarker in future drug development [20, 27, 28]. Our study had only nine patients with the inflamed phenotype limiting further comparisons. Reduced risk of death in patients with the immune-excluded phenotype suggests that the cancer-immune phenotype is a potentially useful biomarker for predicting response to ICI.

Our study found no apparent association between PD-L1 expression and non-synonymous TMB, although the prevalence of high PD-L1 expression was observed in the inflamed phenotypes. Further studies are needed to address a combination use of biomarkers in predicting treatment outcomes in patients with mUC to develop effective immuno-oncology-combination regimens.

The limitations of this study include its retrospective nature; results may be biased due to unmeasured confounding variables. Variability in the timing of sample collection, sample age, FFPE sample preparation, and disease stage of specimens may have caused relatively low rates of PD-L1 expression and we cannot rule out the possibility that the samples do not reflect the immune microenvironment just prior to treatment initiation. In addition, comparisons in some subgroups (e.g., the inflamed phenotype) were restricted due to limited patient numbers. Since first-line treatment was chemotherapy, the predictive value of PD-L1 expression and other biomarkers described in this study did not directly reflect response to ICI therapy. Finally, this study involved only 21 sites in Japan and sample size was limited.

In conclusion, in Japanese patients with stage IV UC treated with first-line chemotherapy and second-line ICIs or chemotherapy, high PD-L1 expression was associated with slightly better ORR to second-line or later treatment including pembrolizumab. Although PD-L1 expression status and non-synonymous TMB were non-significant predictors of survival, cancer-immune phenotype may be an important prognostic factor. Future research to evaluate tumor immune status, particularly the cancer-immune phenotype, in addition to PD-L1 expression status and TMB, is needed to develop more effective treatments in patients with mUC.

### Supplementary Information

Below is the link to the electronic supplementary material.

Supplementary file1 (PDF 260 KB)

## Data Availability

The dataset generated/analyzed during the current study are available from the corresponding author upon reasonable request.
