# Supplementary material for: Tumor immune microenvironment and clinical outcomes in stage IV urothelial cancer: YODO study
Source: Int J Clin Oncol. 2023 Jul 27;28(10):1398–410. doi: 10.1007/s10147-023-02386-y (PMC10543076; doi:10.1007/s10147-023-02386-y)
Supplement: Supplementary file 1 — Supplementary file1 (PDF 260 KB) [file 10147_2023_2386_MOESM1_ESM.pdf]

Tumor Immune Microenvironment and Clinical Outcomes with Chemotherapy and Immune Checkpoint Inhibitor sequential treatment for Stage IV Urothelial Cancer: YODO Study

International Journal of Clinical Oncology

Supplementary Materials

Authors: Hiroyuki Nishiyama[1], Toyonori Tsuzuki[2], Chikara Ohyama[3], Hideyasu Matsuyama[4], Kenta Shinozaki[5], Yuko Hayashi[5], Nobuya Hayashi[5], Ryo Koto[5], Eisei Shin[5], Osamu Ogawa[6]*

Affiliations: [1]Department of Urology, University of Tsukuba, 2-1-1 Amakubo, Tsukuba, Ibaraki, 305-8576, Japan; [2]Department of Surgical Pathology, Aichi Medical University, 1-1 Yazakokarimata, Nagakute, Aichi, 480-1195, Japan; [3]Department of Urology, Hirosaki University, 5 Zaifu-cho, Hirosaki, Aomori, 036-8562, Japan; [4]Department of Urology, Graduate School of Medicine, Yamaguchi University, 1-1-1 MinamiKogushi, Ube, Yamaguchi, 755-8505, Japan; [5]AstraZeneca K.K., 3-1 Ofukacho, Kita-ku, Osaka, 530-0011, Japan; [6]Department of Urology, Japanese Red Cross Otsu Hospital, 1-1-35 Nagara, Otsu, Shiga, 520-8511, Japan.

***Corresponding author:** Osamu Ogawa, MD, PhD

Email: osamuogawa1219@gmail.com

Supplementary Fig S1 Patient deposition

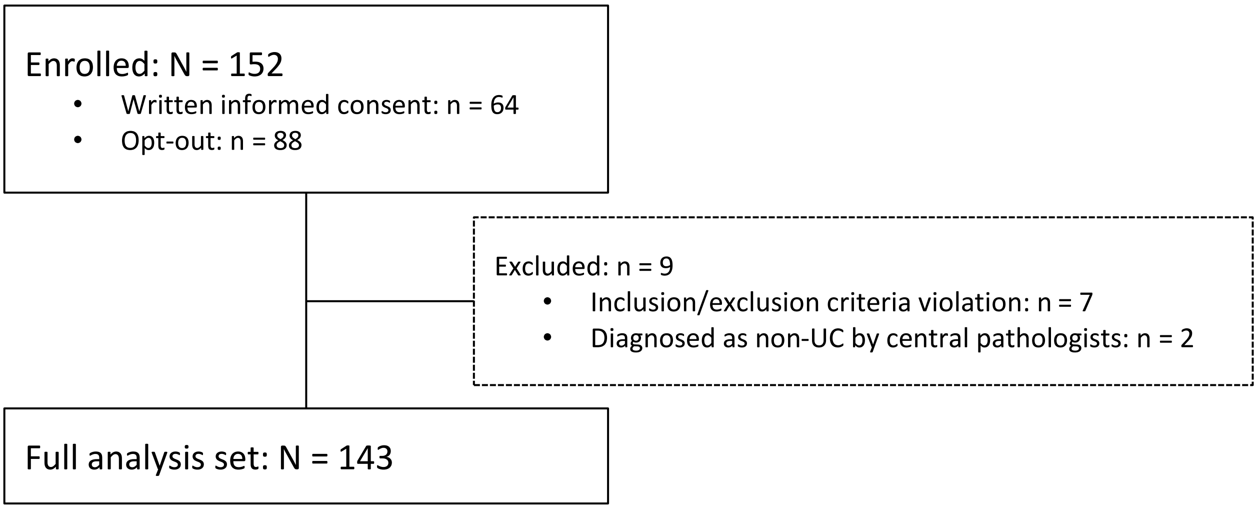

non-UC non-urothelial cancer

Supplementary Table S1 Cancer-immune phenotypes and their distribution by programmed death-ligand 1 expression and the prevalence of non-synonymous tumor mutational burden

	**All (N = 143)**	**PD-L1 expression**		**Non-synonymous TMB**	
		**High (n = 42)**	**Low/negative (n = 101)**	**High (n = 48)**	**Low (n = 95)**
Cancer-immune phenotype, n (%)					
Immune-desert	90 (62.9)	11 (26.2)	79 (78.2)	26 (54.2)	64 (67.4)
Immune-excluded	44 (30.8)	23 (54.8)	21 (20.8)	19 (39.6)	25 (26.3)
Inflamed	9 (6.3)	8 (19.0)	1 (1.0)	3 (6.3)	6 (6.3)

PD-L1 programmed death-ligand 1, *TMB* tumor mutational burden

Supplementary Table S2 Pre-therapy laboratory data and prognostic covariates by programmed death-ligand 1 expression

**Parameter, n (%)**	**All (N = 143)**	**PD-L1 expression**	
		**High (n = 42)**	**Low/negative (n = 101)**
Creatinine clearance			
<60 mL/min	86 (60.1)	20 (47.6)	66 (65.3)
≥60 mL/min	50 (35.0)	19 (45.2)	31 (30.7)
Missing	7 (4.9)	3 (7.1)	4 (4.0)
Serum albumin			
<3.5 g/dL	43 (30.1)	15 (35.7)	28 (27.7)
≥3.5 g/dL	87 (60.8)	25 (59.5)	62 (61.4)
Missing	13 (9.1)	2 (4.8)	11 (10.9)
C-reactive protein			
≤1 mg/L	84 (58.7)	19 (45.2)	65 (64.4)
>1 mg/L	44 (30.8)	17 (40.5)	27 (26.7)
Missing	15 (10.5)	6 (14.3)	9 (8.9)
WBC count			
<8,000/μL	100 (69.9)	24 (57.1)	76 (75.2)
≥8,000/μL	37 (25.9)	17 (40.5)	20 (19.8)
Missing	6 (4.2)	1 (2.4)	5 (5.0)
mGPS			
0	84 (58.7)	19 (45.2)	65 (64.4)
1	14 (9.8)	5 (11.9)	9 (8.9)
2	28 (19.6)	11 (26.2)	17 (16.8)
Missing	17 (11.9)	7 (16.7)	10 (9.9)
NLR			
<500	104 (72.7)	28 (66.7)	76 (75.2)
≥500	24 (16.8)	10 (23.8)	14 (13.9)

Missing	15 (10.5)	4 (9.5)	11 (10.9)
PLR			
<15,000	50 (35.0)	18 (42.9)	32 (31.7)
≥15,000 to ≤30,000	60 (42.0)	15 (35.7)	45 (44.6)
>30,000	18 (12.6)	5 (11.9)	13 (12.9)
Missing	15 (10.5)	4 (9.5)	11 (10.9)
Prognostic index			
0	84 (58.7)	19 (45.2)	65 (64.4)
1	32 (22.4)	10 (23.8)	22 (21.8)
2	13 (9.1)	8 (19.0)	5 (5.0)
Missing	14 (9.8)	5 (11.9)	9 (8.9)
ECOG performance status			
0	40 (28.0)	8 (19.0)	32 (31.7)
1	25 (17.5)	6 (14.3)	19 (18.8)
2	1 (0.7)	0	1 (1.0)
3	3 (2.1)	1 (2.4)	2 (2.0)
Missing	74 (51.7)	27 (64.3)	47 (46.5)

ECOG Eastern Cooperative Oncology Group, *mGPS* modified Glasgow prognostic score, *NLR* neutrophil-to-lymphocyte ratio, *PD-L1* programmed death-ligand 1, *PLR* platelet-to-lymphocyte ratio, *WBC* white blood cell

Supplementary Table S3 Pre-therapy laboratory data and prognostic covariates by non-synonymous tumor mutational burden

**Parameter, n (%)**	**All (N = 143)**	**Non-synonymous TMB**	
		**High (n = 48)**	**Low (n = 95)**
Creatinine clearance			
<60 mL/min	86 (60.1)	27 (56.3)	59 (62.1)
≥60 mL/min	50 (35.0)	18 (37.5)	32 (33.7)
Missing	7 (4.9)	3 (6.3)	4 (4.2)
Serum albumin			
<3.5 g/dL	43 (30.1)	19 (39.6)	24 (25.3)
≥3.5 g/dL	87 (60.8)	25 (52.1)	62 (65.3)
Missing	13 (9.1)	4 (8.3)	9 (9.5)
C-reactive protein			
≤1 mg/L	84 (58.7)	30 (62.5)	54 (56.8)
>1 mg/L	44 (30.8)	13 (27.1)	31 (32.6)
Missing	15 (10.5)	5 (10.4)	10 (10.5)
WBC count			
<8,000/μL	100 (69.9)	33 (68.8)	67 (70.5)
≥8,000/μL	37 (25.9)	14 (29.2)	23 (24.2)
Missing	6 (4.2)	1 (2.1)	5 (5.3)
mGPS			
0	84 (58.7)	30 (62.5)	54 (56.8)
1	14 (9.8)	4 (8.3)	10 (10.5)
2	28 (19.6)	9 (18.8)	19 (20.0)
Missing	17 (11.9)	5 (10.4)	12 (12.6)
NLR			
<500	104 (72.7)	35 (72.9)	69 (72.6)
≥500	24 (16.8)	9 (18.8)	15 (15.8)

Missing	15 (10.5)	4 (8.3)	11 (11.6)
PLR			
<15,000	50 (35.0)	19 (39.6)	31 (32.6)
≥15,000 to ≤30,000	60 (42.0)	21 (43.8)	39 (41.1)
>30,000	18 (12.6)	4 (8.3)	14 (14.7)
Missing	15 (10.5)	4 (8.3)	11 (11.6)
Prognostic index			
0	84 (58.7)	30 (62.5)	54 (56.8)
1	32 (22.4)	9 (18.8)	23 (24.2)
2	13 (9.1)	4 (8.3)	9 (9.5)
Missing	14 (9.8)	5 (10.4)	9 (9.5)
ECOG performance status			
0	40 (28.0)	14 (29.2)	26 (27.4)
1	25 (17.5)	6 (12.5)	19 (20.0)
2	1 (0.7)	0	1 (1.1)
3	3 (2.1)	0	3 (3.2)
Missing	74 (51.7)	28 (58.3)	46 (48.4)

ECOG Eastern Cooperative Oncology Group, *mGPS* modified Glasgow prognostic score, *NLR* neutrophil-to-lymphocyte ratio, *PLR* platelet-to-lymphocyte ratio, *TMB* tumor mutational burden, *WBC* white blood cell

Supplementary Table S4 Pre-therapy laboratory data and prognostic covariates by cancer-immune phenotype

Parameter, n (%)	All (N = 143)	Cancer-immune phenotype		
		**Immune-desert (n = 90)**	**Immune-excluded (n = 44)**	**Inflamed (n = 9)**
Creatinine clearance				
<60 mL/min	86 (60.1)	60 (66.7)	24 (54.5)	2 (22.2)
≥60 mL/min	50 (35.0)	28 (31.1)	16 (36.4)	6 (66.7)
Missing	7 (4.9)	2 (2.2)	4 (9.1)	1 (11.1)
Serum albumin				
<3.5 g/dL	43 (30.1)	29 (32.2)	12 (27.3)	2 (22.2)
≥3.5 g/dL	87 (60.8)	54 (60.0)	27 (61.4)	6 (66.7)
Missing	13 (9.1)	7 (7.8)	5 (11.4)	1 (11.1)
C-reactive protein				
≤1 mg/L	84 (58.7)	56 (62.2)	23 (52.3)	5 (55.6)
>1 mg/L	44 (30.8)	28 (31.1)	14 (31.8)	2 (22.2)
Missing	15 (10.5)	6 (6.7)	7 (15.9)	2 (22.2)
WBC count				
<8,000/μL	100 (69.9)	65 (72.2)	29 (65.9)	6 (66.7)
≥8,000/μL	37 (25.9)	23 (25.6)	12 (27.3)	2 (22.2)
Missing	6 (4.2)	2 (2.2)	3 (6.8)	1 (11.1)
mGPS				
0	84 (58.7)	56 (62.2)	23 (52.3)	5 (55.6)
1	14 (9.8)	9 (10.0)	4 (9.1)	1 (11.1)
2	28 (19.6)	18 (20.0)	9 (20.5)	1 (11.1)
Missing	17 (11.9)	7 (7.8)	8 (18.2)	2 (22.2)
NLR				

<500	104 (72.7)	67 (74.4)	30 (68.2)	7 (77.8)
≥500	24 (16.8)	16 (17.8)	7 (15.9)	1 (11.1)
Missing	15 (10.5)	7 (7.8)	7 (15.9)	1 (11.1)
PLR				
<15,000	50 (35.0)	27 (30.0)	17 (38.6)	6 (66.7)
≥15,000 to ≤30,000	60 (42.0)	43 (47.8)	15 (34.1)	2 (22.2)
>30,000	18 (12.6)	13 (14.4)	5 (11.4)	0
Missing	15 (10.5)	7 (7.8)	7 (15.9)	1 (11.1)
Prognostic index				
0	84 (58.7)	56 (62.2)	23 (52.3)	5 (55.6)
1	32 (22.4)	22 (24.4)	9 (20.5)	1 (11.1)
2	13 (9.1)	6 (6.7)	6 (13.6)	1 (11.1)
Missing	14 (9.8)	6 (6.7)	6 (13.6)	2 (22.2)
ECOG performance status				
0	40 (28.0)	25 (27.8)	12 (27.3)	3 (33.3)
1	25 (17.5)	16 (17.8)	7 (15.9)	2 (22.2)
2	1 (0.7)	1 (1.1)	0	0
3	3 (2.1)	3 (3.3)	0	0
Missing	74 (51.7)	45 (50.0)	25 (56.8)	4 (44.4)

ECOG Eastern Cooperative Oncology Group, *mGPS* modified Glasgow prognostic score, *NLR* neutrophil-to-lymphocyte ratio, *PLR* platelet-to-lymphocyte ratio, *WBC* white blood cell

Supplementary Table S5 Participating physicians and affiliations[a]

Toru Harabayashi (National Hospital Organization Hokkaido Cancer Center)
Atsushi Takahashi (Hakodate Goryoukaku Hospital)
Yasuhiro Hashimoto (Hirosaki University Hospital)
Akihiro Ito (Tohoku University Hospital)
Kojima Takahiro (University of Tsukuba Hospital)
Hiroyuki Fujimoto (National Cancer Center Hospital)
Kazuki Kobayashi (Yokosuka Kyosai Hospital)
Hideaki Miyake (Hamamatsu University Hospital)
Takuya Koie (Gifu University Hospital)
Makito Miyake (Nara Medical University Hospital)
Haruhito Azuma (Osaka Medical and Pharmaceutical University Hospital)
Masao Tsujihata (Osaka Rosai Hospital)
Mutsushi Kawakita (Kobe City Medical Center General Hospital)
Akito Terai (Kurashiki Central Hospital)
Shuichi Morizane (Tottori University Hospital)
Hiroaki Matsumoto (Yamaguchi University Hospital)
Tadahiko Kikugawa (Ehime University Hospital)
Hideo Fukuhara (Kochi Medical School Hospital)
Akira Yokomizo (Harasanshin Hospital)
Hideki Sakai (Nagasaki University Hospital)
Naoki Terada (Faculty of Medicine, University of Miyazaki Hospital)

[a]At the time of study completion
